# Transcriptomic analysis of cyanobacterial alkane overproduction reveals stress-related genes and inhibitors of lipid droplet formation

**DOI:** 10.1099/mgen.0.000432

**Published:** 2020-09-17

**Authors:** Daisy B. Arias, Kevin A. Gomez Pinto, Kerry K. Cooper, Michael L. Summers

**Affiliations:** ^1^​ California State University Northridge, 18111 Nordhoff St, Northridge, CA 91330, USA; ^2^​ University of Arizona, 1117 E. Lowell St, Tucson, AZ 85721, USA

**Keywords:** alkane, lipid droplets, microviridin

## Abstract

The cyanobacterium *Nostoc punctiforme* can form lipid droplets (LDs), internal inclusions containing triacylglycerols, carotenoids and alkanes. LDs are enriched for a 17 carbon-long alkane in *N. punctiforme *, and it has been shown that the overexpression of the *aar* and *ado* genes results in increased LD and alkane production. To identify transcriptional adaptations associated with increased alkane production, we performed comparative transcriptomic analysis of an alkane overproduction strain. RNA-seq data identified a large number of highly upregulated genes in the overproduction strain, including genes potentially involved in rRNA processing, mycosporine-glycine production and synthesis of non-ribosomal peptides, including nostopeptolide A. Other genes encoding helical carotenoid proteins, stress-induced proteins and those for microviridin synthesis were also upregulated. Construction of *N. punctiforme* strains with several upregulated genes or operons on multi-copy plasmids resulted in reduced alkane accumulation, indicating possible negative regulators of alkane production. A strain containing four genes for microviridin biosynthesis completely lost the ability to synthesize LDs. This strain exhibited wild-type growth and lag phase recovery under standard conditions, and slightly faster growth under high light. The transcriptional changes associated with increased alkane production identified in this work will provide the basis for future experiments designed to use cyanobacteria as a production platform for biofuel or high-value hydrophobic products.

## Data Summary

The quantitative transcriptomic (RNA-seq) data including the raw sequencing reads, individual sample gene expression levels, and normalized abundance gene expression levels have been deposited in the National Center for Biotechnology Information (NCBI) Gene Expression Omnibus database under the accession numbers: GSE140625, GSM4175947, GSM4175948, GSM4175949, GSM4175950, GSM4175951 and GSM4175952. The authors confirm that all supporting data and protocols have been provided within the article or in a supplementary data file.

**Impact Statement**

*
Nostoc punctiforme* is a filamentous nitrogen-fixing cyanobacterum that forms internal lipid droplets containing diacylglycerols, carotenoids and 17 carbon-long alkanes. All cyanobacteria can produce small amounts of alkanes, well below the production levels found in oleaginous algae, but their physiological function remains elusive. To better understand cellular adaptations associated with overproduction of alkanes that could lead to use of cyanobacteria as a feedstock for biodiesel or for production of hydrophobic biomolecules, we determined the transcriptomic changes associated with alkane overproduction. We found many highly upregulated genes in the overproduction strain involved with cellular stress and the production of unique secondary metabolites. When we reintroduced upregulated genes and operons, several were found to reduce alkane overproduction, and one operon resulted in complete loss of lipid droplet formation. These results indicate potential negative regulators of alkane production and lipid droplet formation. The results of this study are useful in understanding the cellular response to alkane overproduction, which is not only important for the development of cyanobacteria as a feedstock for biofuels, but also as production platforms for high-value hydrophobic biomolecules.

## Introduction

The use of fossil fuels is an unsustainable method of energy production with negative long-term environmental impacts, necessitating progress on alternative fuels that are compatible with existing technologies. Production of biofuels by photosynthetic organisms is one such alternative with great potential to recycle carbon dioxide released by burning fossil fuels back into energy-rich compounds. Bacterial- or algal-generated lipids can be hydrolyzed to fatty acids and glycerol and then converted to biodiesel by methylating the fatty acids to form fatty acid methyl esters. Lipid production is induced in algae by nitrogen starvation, which triggers the accumulation of photosynthate into triacylglyerols concentrated in lipid droplets (LDs). Cyanobacteria such as *Nostoc punctiforme* are one type of bacteria that can produce LDs, but more research is needed to increase their level of production. LDs in cyanobacteria increase during stationary phase, and unlike algae, do not require nitrogen starvation for their production [1]. Cyanobacterial LDs such as those found in *N. punctiforme* are unique in that they also contain ~17 % alkanes, relative to total extracted fatty acids, of a length typically found in jet or diesel fuel mixed with triacylglyerols [1], giving added value to the use of cyanobacteria for biofuel production.

C15-C19 alka(e)ne production is common among cyanobacteria [2, 3], and synthesis occurs via one of two different pathways [4]. The first pathway uses a multi-domain containing polyketide synthesase enzyme (Ole) catalyzing 2-carbon elongation of fatty acyl-acyl carrier protein (acyl-ACP) and subsequent decarboxylation to produce odd carbon alka(e)ns one carbon longer than the C16 and C18 carbon-long fatty acids typically present in cyanobacteria [5]. The second alka(e)ne-producing pathway starts from the same acyl-ACP precursor, but uses the sequential action of two enzymes that first activate and then remove a formyl group to produce odd length alka(e)nes one carbon shorter than the fatty acid substrate [6, 7]. All cyanobacteria with a sequenced genome possess one of these pathways for alkane production, indicating a conserved physiological importance [3].

Recent studies have been conducted to determine the role of alkanes in cyanobacteria. Both *Synechocystis* sp. PCC 6803 and *Synechococcus* sp. PCC 7002 mutants deficient in alkane production exhibited reduced growth, as well as enlarged cell size and increased division defects, likely caused by reduced membrane flexibility and curvature [8]. Berla *et al*. [9] showed that a *Synechocystis* 6803 alkane mutant grew poorly at low temperatures and had enhanced cyclic electron flow, especially at low temperatures. Thus it appears that alkanes may be essential for proper membrane fluidity, and aid in regulation of the ATP : NADPH energy/reductant balance required for cyanobacterial adaptation to daily environmental changes.

In *N. punctiforme,* Npun_R1711 and Npun_R1710 encode acyl-ACP reductase (Aar), and aldehyde deformylating oxygenase (Ado), respectively, which act sequentially to produce only 17 carbon-long alkanes [10]. Orthologues of these enzymes from other cyanobacteria, especially when expressed in an *Escherichia coli* host*,* produce alkanes and alkenes in a variety of lengths [6], indicating the possibility of tailoring alka(e)ne production to fit economic needs. When *aar* and *ado* were present on a multi-copy plasmid along with the putative lipase Npun_F5141, *N. punctiforme* displayed a 16-fold increase in C17 alkane production, which also stimulated increased LD formation [10]. Alkanes were found to be highly enriched in LDs, and not in pelleted membranes and cell debris, leading to the hypothesis that increased LDs were formed as a way to sequester excess alkanes, and keep them from interfering with the normal functioning of photosynthetic and cell membranes [1].

To better understand the physiological response to increased hydrophobic compound production in cyanobacteria, we initiated a transcriptomic study to identify changes associated with increased alkane production. Although alkanes are relatively low-value products in our current economy, the transcriptional response, especially of upregulated genes related to stress responses, may be of benefit to future researchers wishing to optimize the production of higher-value hydrophobic compounds in the future. Just as was found for alkanes [10], overproduced high-value compounds or metabolites produced in *N. punctiforme* will likely partition into LDs, enabling separation from other cell components by floatation following cell lysis. In addition, it may be possible to combine the data presented here with advances in alkane production using metabolic engineering [11–17], or to enable shorter-chain alkane production in cyanobacteria for direct production of biofuels that can be secreted into the media to make production more economically viable [18]. Overall, these results will aid studies to further utilize cyanobacteria as a production platform for alkanes and other hydrophobic compounds in the future.

## Methods

### Bacterial growth conditions

*N. punctiforme* PCC 73102 (ATCC 29133) liquid cultures were grown in 125 ml Erlenmeyer flasks containing 50 ml of Allen and Arnon media (AA) diluted fourfold (AA/4) [19] supplemented with 5 mM 3-(*N*-morpholino)propanesulfonic acid (MOPS), 2.5 mM NH_4_Cl, 2.5 mM KNO_3_ and 2.5 mM NaNO_3_ (collectively known as MAN). Plate AA media were not diluted and contained MAN and 1 % Noble agar. Cultures harbouring pSCR119-based plasmids were supplemented with neomycin at a final concentration of 10 µg ml^−1^. Flasks were incubated under a white fluorescent light (12–15 μmol photons m^2^ sec^−1^ between 400–700 nm) at 25 °C and shaken at 120 r.p.m. Plates were statically grown at the same illumination and temperature in a CO_2_-enriched (5000 p.p.m.) growth chamber. Altered parameters were temperature (15 °C) for cold growth experiments, and illumination (110 μmol photons m^2^ s^−1^) for high-light experiments. *E. coli *, DH5-α MCR, was grown in Luria–Bertani (LB) broth and agar plates at 37 °C using 30 µg ml^−1^ kanamycin for plasmid selection.

### Plasmid and strain construction

The two-gene Npun_F1710/11 expression (2 g) plasmid was made by PCR-amplifying adjacent genes Npun_R1711 and Npun_R1710 and the upstream intergenic region, and subsequent cloning into pSCR119, a shuttle plasmid capable of replication in both *E. coli* and *N. punctiforme* as described previously [10]. PCR fragments of various genes or operons found to be upregulated during comparative transcriptomic analysis in the 2 g strain were generated using the appropriate upstream P1 and downstream P2 primers (Table S1) using Herculase II Fusion DNA Polymerase (Aligent) or Phusion High Fidelity Polymerase (Thermo Scientific), and cloned into the KpnI/SacI sites of pSCR119 to create the ‘single-gene’ plasmids. These same fragments were similarly cloned into a previously constructed ‘three-gene’ plasmid consisting of pSCR119 containing Npun_F1710/11 and Npun_F5141 [10] to create a set of ‘four-gene’ plasmids. The pSCR119 plasmid does not contain a promoter to drive transcription of inserted DNA and so upstream intergenic regions were included in all gene inserts to allow transcription from native promoters. All inserted genes in four-gene plasmids were cloned in the same transcriptional orientation, and downstream from the existing three-genes. All plasmids were verified by Sanger sequencing and transformed into *N. punctiforme* by electroporation [20].

### RNA preparation, RNA-seq library preparation and sequencing

Triplicate cultures of log-phase wild-type plasmid-only control (WTC) and 2 g strains were harvested by a 2 min centrifugation at 6000 ***g***. The pellet was suspended in 700 µl of media prior to flash freezing and storage at −80 °C. RNA was harvested as described previously [21]. The samples were then further purified using a RNeasy Mini Kit (Qiagen) with on-column DNase treatment. Samples were aliquoted and stored at −80 °C until used. Five micrograms of each sample was cleaned and concentrated further using an RNA Clean and Concentrator-5 kit (Zymo). rRNA depletion was then performed using Terminator 5′-Phosphate-Dependent Exonuclease (Epicentre) following the manufacturer’s protocol using buffer A.

Strand-specific RNA libraries were prepared and barcoded using the NEBNext Ultra Directional RNA Library Prep kit for Illumina and NEBNext Multiplex Oligos for Illumina (New England Biolabs) per the manufacturer’s instructions. Library quality and size distribution was determined using an Experion 1K DNA analysis kit (Bio-Rad), and then each cDNA library was quantified using a Qubit Fluorometer and dsDNA High Sensitivity reagents (Invitrogen) according to the manufacturer’s instructions. Each library was quantified via qPCR prior to pooling using the Library Quantification kit for Illumina platforms (KAPA), and then each library was normalized to 10 nM and pooled for sequencing. The multiplexed pooled libraries containing all six samples were then sequenced as single-end 100 bp reads on an Illumina HiSeq 2500 system at the UC Irvine Genomics High-Throughput Facility.

### Transcriptomic data analysis

Initially all the reads from each sample were trimmed for Illumina adapters and low-quality sections (<Q20) or N bases using Trimmomatic (v0.39) [22], and then the reads were quality-filtered (>Q30 across >90 % of read) with Galaxy Filter FASTQ (v1.0.0) [23] to eliminate all low-quality reads prior to analysis. Next, each sample’s reads were aligned to the *N. punctiforme* complete genome sequence (GenBank accession numbers: CP001037.1, CP001038.1, CP001039.1, CP001040.1, CP001041.1 and CP001042.1) using Tophat (v2.0.13) [24], and then individual sample gene expression levels were quantified based on fragments per kilobase of transcript per million mapped reads (FPKM) using Cufflinks (v2.2.1) [25]. Finally, all the individual sample gene expression levels from Cufflinks were merged together using Cuffmerge (v1.0.0), and the WTC transcriptome was compared to the LD/alkane overproducer (2 g) transcriptome using Cuffdiff (v2.2.1) [26]. Those genes with statistically significant differences (*q*-value <0.05 or *P*-value <0.004) between the WTC and 2 g strains were determined. The quantitative transcriptomic (RNA-seq) data. including the raw sequencing reads, individual sample gene expression levels and normalized abundance gene expression levels, have been deposited in the National Center for Biotechnology Information (NCBI) Gene Expression Omnibus database under the accession numbers: GSE140625, GSM4175947, GSM4175948, GSM4175949, GSM4175950, GSM4175951 and GSM4175952.

### RT-qPCR analysis

RNA was independently extracted from cells grown under the same growth condition as described previously for transcriptomic (RNA-seq) analysis. First-strand cDNAs were synthesized using SuperScript II Reverse Transcriptase (Invitrogen) with gene-specific P2 primers (Table S1). qPCR was performed following multiplex reverse transcription using FastStart Universal SYBR Green Master with ROX (Roche) using five fivefold serial dilutions of each cDNA sample to determine PCR efficiency and favourable dilution factor. The manufacturer’s protocol was modified for 20 µl reactions. Four technical replicates per duplicate or triplicate biological replicates were subjected to qPCR. The gene-specific primer sets used for qPCR are listed in Table S1 (available in the online version of this article).

### Lipid extraction and analysis

For alkane and fatty acid analysis from whole cells and LDs, triplicate samples of exponential and stationary cultures were subjected to lipid extraction, saponification and methyl esterification to produce fatty acid methyl esters (FAMEs) as described previously [10]. LDs were harvested as described previously [1]. Due to the unavailability of 12 % BCl_3_ in methanol from the suppliers used to prepare methyl esters for the above samples, LD samples were converted to FAMEs using the protocol of Ichihara and Fukubayashi [27].

Analysis of all FAMEs was performed using a SHIMADZU/QP2010S gas chromatography mass spectrometer (GC-MS). The GC was equipped with a SHRX1-5MS column (30 m × 0.25 mm I.D., 0.25 µm film thickness). The oven temperature was held at 180 °C for 1 min and increased to 300 °C at 12 °C min^−1^, with the final temperature of 300 °C then maintained for 2 min. Helium was used as the carrier gas and 1 µl of sample was injected in split mode (1 : 75). MS detector voltage was set at 0.25 kV, and the samples were identified using NIST11 and NIST11s libraries. FAME standards (RESTEK cat #35066) were used to confirm identifications.

### LD staining and analysis

Screening strains for altered LD phenotypes was accomplished by fluorescence microscopy using the non-toxic fluorescent nonpolar dye BODIPY 505/515 (Molecular Probes cat #D3921). One microlitre of a working stock solution (235 µg ml^−1^ in DMSO) was added to 20 µl of a cell culture and incubated for 5 min in the dark before the observation of LDs as a wet mount on a Zeiss Axiolab epifluorescence microscope equipped with a blue (475±20 nm) excitation filter and a green (535±23 nm) emission filter.

## Results and discussion

### Comparative transcriptomics and verification of results

**Fig. 1.**
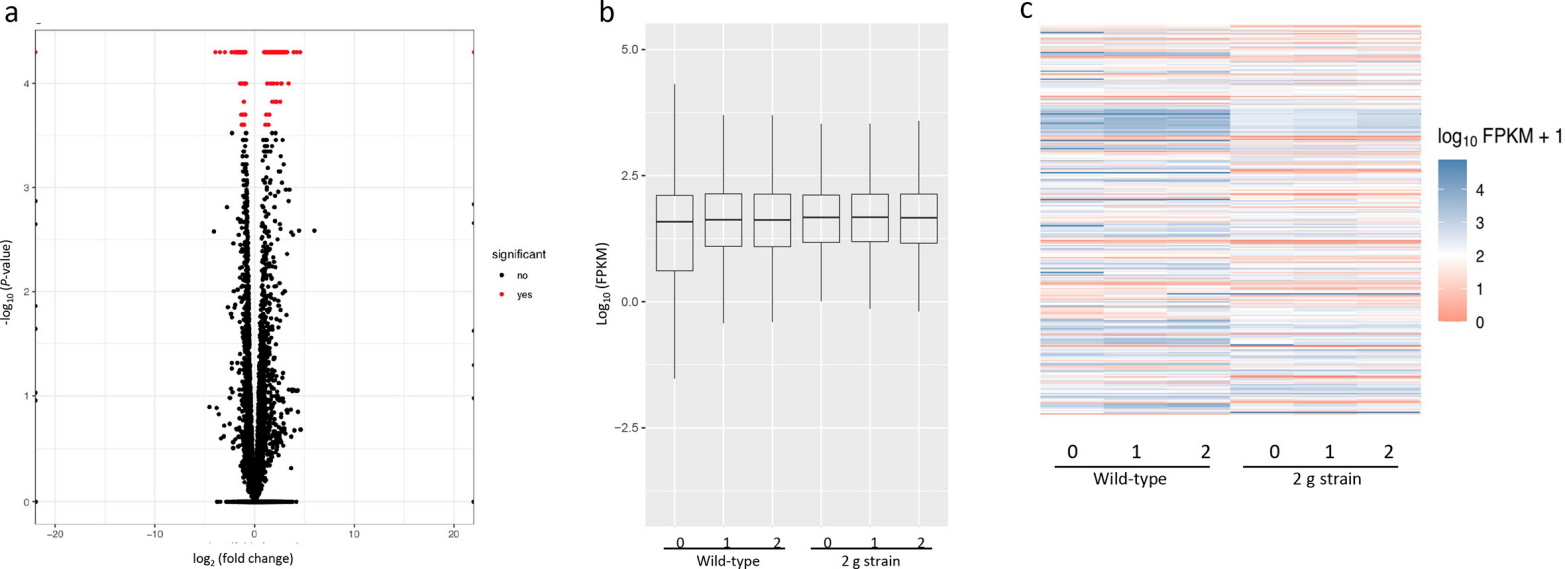
Transcriptomic analysis of alkane overproduction in *N. punctiforme *. (a) Volcano plot showing all significantly regulated genes in the 2 g alkane overproduction strain. (b) Boxplot representation of normalized reads (fragments per kilobase of transcript per million mapped reads; FPKM) for each replicate. Box indicates upper and lower quartile, the bar indicates the median, and the whiskers indicate the highest and lowest values excluding outliers, indicated by circles. (c) Heat map representation of gene expression with twofold differences for each replicate. All figures were generated using the CummeRbund package (v2.30.0) for R.

Comparative transcriptomic analysis of the samples identified 421 genes that were significantly regulated between the wild-type plasmid-only control strain and the alkane overexpressing ‘2 g strain’ bearing pSCR119 containing Npun_R1710 and Npun_R1711. The volcano plot in Fig. 1a depicts the expression levels of all genes present in the *N. punctiforme* PCC 73102 genome, including significantly altered gene expression (red circles) between the two groups. FPKM values for each replicate were similar and normally distributed, as required for analysis by the Tuxedo suite (Fig. 1b). Using a 2-fold or greater cutoff of significantly expressed genes, we identified 177 upregulated and 121 downregulated genes that responded to overproduction of alkanes. Their expression is visualized as a heat map (Fig. 1c) and a complete list can be found in Tables S2 and S3. Table 1 contains genes mentioned in the text. As an internal control, it was found that Npun_R1710 and Npun_R1711, were upregulated 15- and 23-fold, respectively. This level of induction is in general agreement with the previously found copy number of ~14 copies per chromosome for this plasmid [20].

**Table 1.** Differentially regulated genes mentioned in the text. Adjacent genes are indicated by highlighted text. See Tables S2 and S3 for a complete list of significant differentially regulated genes with a ≥twofold change in expression

Upregulated genes in the 2 g strain			
**Gene**	**Function**	**Fold change**	***P*-value**	***q*-value**
Npun_BF041	AraC family transcriptional regulator	**8.08**	5.00E−05	0.00134573
Npun_F0469	CsbD family stress response protein	**15.00**	5.00E-05	0.00134573
Npun_F1277	Putative signal transduction histidine kinase with PAS/PAC domains	**3.39**	0.00015	0.00346156
Npun_F1545	Protein of unknown function	**2.34**	0.001	0.0156562
Npun_F2181	NosA; nostopepolide A synthesizing amino acid adenylation/condensation NRPS	**5.26**	5.00E−05	0.00134573
Npun_F2183	NosC; nostopepolide A synthesizing amino acid adenylation/condensation NRPS	**7.97**	5.00E−05	0.00134573
Npun_F2184	NosD; nostopepolide A synthesizing amino acid adenylation/condensation NRPS	**5.08**	5.00E−05	0.00134573
Npun_F2189	Putative peptide macrocyclase/ligase, Mvd	**10.56**	0.0001	0.00242941
Npun_F2191	Putative glycolipid lipase	**6.54**	5.00E−05	0.00134573
Npun_F2192	Protein of unknown function	**6.30**	0.0001	0.00242941
Npun_F2818	ABC1 kinase (ABC1K1)	**2.24**	5.00E−05	0.00134573
Npun_F2819	Protein of unknown function	**2.65**	0.004	0.0423216
Npun_F3786	Protein of unknown function	**2.53**	5.00E−05	0.00134573
Npun_F3787	BON domain-containing protein (membrane attachment)	**4.42**	5.00E−05	0.00134573
Npun_F3789	Protein of unknown function	**8.11**	5.00E−05	0.00134573
Npun_F4818	Putative transporter	**4.11**	5.00E−05	0.00134573
Npun_F4819	General stress response domain-containing protein	**18.62**	5.00E−05	0.00134573
Npun_F5451	YcnF-like stress response PRC barrel domain-containing protein	**9.48**	5.00E−05	0.00134573
Npun_F5452	YcnF-like stress response PRC barrel domain-containing protein	**6.65**	5.00E−05	0.00134573
Npun_F5453	Protein of unknown function	**3.65**	5.00E−05	0.00134573
Npun_F5913	Orange carotenoid family protein	**5.91**	0.0007	0.011825
Npun_F6242	Orange carotenoid family protein	**8.39**	5.00E−05	0.00134573
Npun_R0959	CsbD family stress response protein	**5.65**	5.00E−05	0.00134573
Npun_R0971	Hemerythrin domain-containing protein	**5.80**	5.00E−05	0.00134573
Npun_R1332	Protein of unknown function	**63.53**	0.0026	0.0311091
Npun_R3254	CsbD family stress response protein	**2.60**	5.00E−05	0.00134573
Npun_R3425	PKS/NRPS – beta-ketoacyl synthase	**3.31**	5.00E−05	0.00134573
Npun_R3426	PKSNRPS – beta-ketoacyl synthase	**4.12**	5.00E−05	0.00134573
Npun_R3429	PKS/NRPS – condensation domain-containing protein	**2.13**	0.0011	0.0168906
Npun_R3430	PKS/NRPS – beta-ketoacyl synthase	**4.36**	5.00E−05	0.00134573
Npun_R3431	PKS/NRPS – amino acid adenylation domain-containing protein	**3.23**	5.00E−05	0.00134573
Npun_R3432	PKS/NRPS – beta-ketoacyl synthase	**3.89**	5.00E−05	0.00134573
Npun_R3433	PKS/NRPS – amino acid adenylation domain-containing protein	**9.70**	5.00E−05	0.00134573
Npun_R3434	Methyltransferase domain-containing protein	**12.89**	0.0027	0.0318286
Npun_R3435	PKS/NRPS – condensation domain-containing protein	**8.42**	5.00E−05	0.00134573
Npun_R3436	PKS/NRPS – amino acid adenylation domain-containing protein	**5.47**	5.00E−05	0.00134573
Npun_R3438	PKS/NRPS – condensation domain-containing protein	**4.68**	0.00015	0.00346156
Npun_R3440	acyl-CoA dehydrogenase domain-containing protein	**5.43**	0.0007	0.011825
Npun_R3442	3-hydroxyacyl-CoA dehydrogenase	**5.15**	0.0011	0.0168906
Npun_R3445	PKS/NRPS – betaketoacyl synthase	**5.35**	5.00E−05	0.00134573
Npun_R3446	Class III aminotransferase family protein	**8.20**	0.00375	0.0405359
Npun_R3449	Glycosyl transferase family protein	**5.95**	0.00015	0.00346156
Npun_R3450	PKS/NRPS – amino acid adenylation domain-containing protein	**7.26**	5.00E−05	0.00134573
Npun_R3451	Predicted (di)oxygenase family protein	**3.64**	0.0047	0.0471062
Npun_R3452	PKS/NRPS – AMP-dependent synthetase and ligase	**2.10**	5.00E−05	0.00134573
Npun_R4091	SigB2 RNA polymerase sigma factor	**2.05**	0.0004	0.00771465
Npun_R4582	Manganese-containing catalase	**7.51**	0.0006	0.0104908
Npun_R5130	Orange carotenoid family protein	**4.66**	5.00E−05	0.00134573
Npun_R6442	Hypothetical protein	**6.10**	0.0025	0.0303676
Npun_F5066	Predicted non-haem iron protein	**7.00**	0.00035	0.00694031
Npun_R5598	MysC; mycosporine-glycine ligase	**4.16**	0.0021	0.0271031
Npun_R5599	MysB; SAM-dependent DGG O-methyltransferase	**14.40**	0.00285	0.033044
Npun_R5600	MysA; demethyl 4-deoxygadusol (DDG) synthase	**21.84**	0.0026	0.0311091
	**Downregulated genes in the 2 g strain**			
**Gene**	**Function**	**Fold change**	***P*-value**	***q*-value**
Npun_F0518	SpoVK-like vesicle-fusing AAA+-type ATPase	−**1.99**	5.00E−05	0.00134573
Npun_F0996	SigC – RNA polymerase sigma factor	−**2.57**	5.00E−05	0.00134573
Npun_F1653	Magnesium-protoporphyrin IX monomethyl ester cyclase	−**6.87**	0.00155	0.0215364
Npun_F3794	Phycobilisome linker polypeptide CpeC	−**16.93**	5.00E−05	0.00134573
Npun_F3795	Phycobilisome linker polypeptide CpcG2	−**12.81**	0.0002	0.00437916
Npun_F4466	Nitrogen regulatory protein PII	−**2.28**	5.00E−05	0.00134573
Npun_R0279	Universal stress protein domain-containing protein	−**3.40**	5.00E−05	0.00134573
Npun_R0404	Carotenoid binding domain-containing protein	−**11.11**	5.00E−05	0.00134573
Npun_R1304	PadR-like family transcriptional regulator	−**2.43**	0.00245	0.0298208
Npun_R4883	Low temperature-requirement A-like protein	−**2.00**	0.00125	0.0185978

Most highly regulated genes were found to be upregulated rather than downregulated; only 11 downregulated genes displayed greater than 4-fold change, whereas 85 upregulated genes changed 4-fold or more. Genes encoding proteins of unknown function make up around ~29 % of upregulated genes and ~19 % of downregulated genes. To verify the validity of the comparative transcriptomic data, several up and downregulated genes were tested using RT-qPCR on independently isolated RNA (Table 2). The RNA-seq transcriptomic data in general exhibited larger changes in gene expression, but overall the trends and relative changes among those tested were confirmed by RT-qPCR on independently isolated samples.

**Table 2.** Validation of RNA-seq by qPCR. Average fold change from the control strain for 11 selected genes showing 2-fold or higher change in the 2 g alkane overproduction strain were confirmed by independent RT-qPCR. ± standard error, *n*=3 unless indicated by bold numbers where *n*=2. Note that the Npun_F2818-Npun_F2819 qPCR primer set spanned the 26 bp intergenic region between these two genes and was therefore used to measure both

Gene	RT-qPCR	RNA-seq
Npun_F1653	−3.43±0.61	−6.87
Npun_F3794	−8.23±0.71	−16.93
Npun_F3795	−9.04±0.80	−12.81
Npun_F4819	7.99±1.02	18.62
Npun_R1332	21.44±3.24	63.53
Npun_F1545	**1.76±0.38**	2.34
Npun_F2189	**6.86±1.37**	10.56
Npun_F2191	**6.91±1.29**	6.54
Npun_F2818	**2.42±0.50**	2.24
Npun_F2819	**2.42±0.50**	2.65
Npun_R0971	**6.59±1.28**	5.80

### Identification of regulated genes

#### Upregulated genes in the alkane overproduction strain

The most highly upregulated gene in this study encoded a protein of unknown function, Npun_R1332, differentially expressed >60-fold in the 2 g strain. It contains a NYN domain (Nedd4-BP1 and YacP Nuclease; Pfam01936), predicted to be involved in processing tRNA and ribosomal RNAs [28]. The gene encoding ribonuclease 3 in *N. punctiforme *, Npun_R1331, is adjacent to Npu_R1332. In *Oscillatoria acuminate*, an orthologue of this gene is fused to ribonuclease 3 that is involved in the processing of primary rRNA transcripts [29], further supporting a role for Npun_R1332 in ribosomal assembly. To the best of our knowledge, it is unknown if alkanes interfere with ribosomal processing or assembly that might be alleviated by such large increases in transcription for this protein. The only other predicted RNA processing protein, Npun_R2514, was only twofold upregulated in the 2 g strain.

The second most upregulated gene in the 2 g strain, Npun_R5600, is one of three adjacent genes (Npun_R5600-5599-5598; *mysA-mysB-mysC*) upregulated 22-, 14- and 4-fold, respectively, used in the production of mycosporine-glycine. Mycosporines are UV-absorbing secondary metabolites, and mycosporine-glycine has been found to quench singlet oxygen and absorb UV light to protect against photodamage [30]. These small water-soluble cyclic molecules can also function as compatible solutes and nitrogen storage compounds, and for defence against thermal, desiccation and other stress conditions [31]. Npun_R5600 encodes demethyl 4-deoxygadusol (DDG) synthase, a sugar phosphate cyclase forming DDG from sedoheptulose 7-phosphate, and Npun_R5599 encodes a SAM-dependent O-methyltransferase that catalyses the methylation of DDG [32]. Npun_R5598 encodes a ligase that catalyses the condensation of glycine onto DDG to produce mycosporine-glycine [33]. This response likely indicates that mycosporine-glycine ameliorates stress caused by alkane accumulation in cyanobacteria, and its induction is triggered by alkane-induced membrane or photosynthetic signals that overlap with photodamage.

The third most upregulated gene in the 2 g strain, Npun_F4819, is similar to general stress-induced protein B (GsiB) from *Bacillus subtilis* [34] and was induced ~19-fold in the 2 g strain. The exact function of GsiB-like proteins has not yet been determined; however, an increase of this magnitude implies that this protein may be induced to cope with alkane overproduction and alleviate alkane-induced stress. As such, it may be a good target gene to co-express in order to increase alkane yields and/or cyanobacterial vigour in alkane overexpression strains. The upstream gene, Npun_F4818, encoding a protein of unknown function, showed a fourfold increase in the 2 g strain, and has four predicted transmembrane domains with some structural similarity to transporter proteins. The association of these two genes is not conserved in other organisms in the STRING interaction database, indicating that they may have non-associated functions [29].

Several proteins belonging to the CsbD family of stress response proteins were upregulated in the 2 g strain. *csbD* gene expression has been shown to be induced as part of the sigma B general stress response in *B. subtilis,* although the protein’s exact role in stress response is unknown [35]. CsbD-like proteins Npun_F0469, Npun_R0959 and Npun_R3254 increased 15-, 5.7- and 2.6-fold in the 2 g strain, respectively. Sig B2 encoded by Npun_R4091 was also upregulated twofold in the 2 g strain and, in line with control by a sigma factor in *Bacillus,* may represent the potential regulator for their transcription. It is interesting to note that all three CsbD family proteins co-occur in genomes of other bacteria along with AvaK-like PRC-barrel domain-containing proteins Npun_F5452 and Npun_F5451 in the STRING database [29]. These AvaK homologues are also upregulated in the 2 g strain and are presented below.

Three upregulated genes encode proteins containing a conserved N-terminal carotenoid-binding domain (NTD) similar to that found in the orange carotenoid-binding protein (OCP). All three, however, lack the C-terminal domain that regulates NTD binding to the phycobilisome that results in quenching of excess excitation energy during high light stress. This NTD-only protein class has been termed ‘Helical Carotenoid Proteins’ (HCPs), and occur commonly in cyanobacteria [36]. Orthologous HCPs have been studied in *Nostoc* sp. strain PCC 7120, and some have defined functions. Npun_F5913 and Npun_R5130 induced 5.9- and 4.7-fold in the 2 g strain, are orthologues of All3221, and Alr4783 has been found to quench singlet oxygen [37]. The third protein, Npun_F6242, was upregulated 8.4-fold in the 2 g strain and is orthologous to All1123, an HCP with no determined function [37].

A large gene cluster encoding a 19-gene hybrid polyketide synthase (PKS) and non-ribosomal peptide synthesis (NRPS) assembly line referred to as the pks4 gene cluster [38] were upregulated 2- to 13-fold in the 2 g strain. These include Npun_R3425-3426, Npun_R3429-3436, Npun_R3438, 3440, 3442, 3445–46, and Npun_R3449-52. Among these are 13 encoded proteins containing a variety of PKSs and non-ribosomal peptide synthesis (NRPS) domains [39]. These are interspersed with genes encoding predicted dehydrogenases, amino-and glycosyl-transferases and a dioxygenase. The pks4 gene cluster was shown to be expressed in a regular spaced pattern by single or neighbouring cells within a filament using a Npun_R3452-GFP reporter, and this gene cluster was strongly induced when cultures were grown to ultrahigh density [40]. The exported product of the pks2 gene cluster has been shown to affect cellular differentiation [38], although the product of the pks4 gene cluster remains unknown. The bioactive compounds produced by these clusters have a wide range of activities, including cytotoxicity, enzyme inhibition, antibacterial and antifungal agents [41]. It will be interesting to see if these can be extended to alkane tolerance in future work.

A second locus encoding NRPSs upregulated five- to eightfold in the 2 g strain include orthologues of NosA, NosC and NosD, encoded by Npun_F2181, Npun_F2183 and Npun_F2184, respectively. The orthologous proteins in *Nostoc* sp. GSV224 are the non-ribosomal peptide synthases that form the peptide backbone for nostopeptolide A [42]. Interestingly, *nosB* and four additional downstream genes conserved in this locus encoding peptides with polyketide synthase, dehydrogenase, reductase and transporter activities were not upregulated in the 2 g strain. Nostopepolide A was found to be exported into the extracellular polysaccharide matrix, and to be an important regulator of hormogonium development in *N. punctiforme* [43]. The finding of upregulation for only the peptide backbone-forming genes without similar upregulation of the associated transporter may indicate that the peptide backbone likely accumulates in cells for adaptation to alkane stress. We hope future work by others will determine if non-ribosomal peptides or polyketides are capable of sequestering alkanes *in vitro,* providing evidence for a possible mechanism for this adaptation that would explain the upregulation of the associated genes and operons discovered here.

Several upregulated genes with potential direct involvement in ameliorating stress in lipid membranes were identified. Npun_F3787 was upregulated 4.4-fold in the 2 g strain and has an N-terminal signal sequence as identified by SPOCTOPUS [44] as well as a BON domain in the C-terminal portion of the protein. BON domains are thought to bind phospholipids and aid in stabilizing membranes [45], similar to OsmY, an osmotically inducible periplasmic protein providing protection against osmotic shock [46]. This may provide a protective mechanism to cope with increased membrane fluidity caused by alkanes that would interfere with cell and photosynthetic membrane functions. The downstream gene, Npun_F3786, was also upregulated 2.5-fold and encodes a putative signal transduction histidine kinase that may be involved in regulating this stress response.

Several genes involved in microviridin synthesis were upregulated. Microviridins are tricyclic members of ribosomally synthesized and post-translationally modified peptides that act as protease inhibitors. Npun_F2189 was over 10-fold upregulated in the 2 g strain and encodes an ATP-grasp peptide maturases, similar to MvdC (MdnB). This gene is preceded by the precursor peptide MvdA with the amino acid sequence MPTNTVKTVDVVAVPFFARFLEEQATEGTEVPW**T**Y**K**F**PSD**LEDR [47]. Analysis of the novel microviridins N3–N9 from *N. punctiforme* identified a core region in this peptide (double underline above) that contained tricyclic linkages (bold) with a variable 1–6 amino acid extension (single underline) instead of acylation normally present in microviridins [40]. Normally, MvdC and the downstream MvdD (MdnC), encoded by Npun_F2190, act to form the intramolecular lactam and lactone linkages, respectively, in the MvdA peptide, resulting in a tricyclic peptide with a unique cage-like structure. However, the downstream gene encoding MvdD was not upregulated, indicating that an altered form of this microviridin with a single amide and lacking ester bonds may be formed due to alkane overproduction. The next two downstream genes – Npun_F2191, encoding a putative lipase, and Npun_F2192, encoding a predicted transmembrane protein of unknown function – were both induced ~sixfold in the 2 g strain. As presented below, the presence of these four genes (Npun_F2189–2192) on a multi-copy plasmid resulted in loss of LD production. The reason for this unclear, but the core region of microviridins N3–N9 contains 43 % hydrophobic and 21 % neutral amino acids. We speculate that these microviridins may be sequestering hydrophobic compounds normally found in LDs when overexpressed, thus interfering with LD formation. Our results differ from the RNA-seq results for high-density growth used to induce production of microviridins N3–N9. A downstream ABC transporter encoded by Npun_F2193 with 61/79 % identity/similarity to MdnE, thought to export this peptide, was not upregulated in the 2 g strain. This indicates a potential for intracellular localization of this microviridin, similar to the results for nostopeptolide A mentioned above.

Three adjacent genes encoding PRC barrel domain-containing proteins were induced 3.6–9.5-fold in the 2 g strain. These include the akinete marker protein, AvaK (Npun_F5452), and its adjacent upstream and downstream genes. AvaK and its upstream paralogous protein, Npun_F5451, were also found to accumulate after butachlor exposure in three different cyanobacteria, and were hypothesized to be involved in tolerance to stress associated with exposure to this herbicide [48]. Both contain a PRCH domain (photosynthetic reaction centre subunit H) that functions to regulate electron passage between quinones in the photosynthetic reaction centre of purple bacteria [49]. Transcriptional upregulation in the 2 g strain may therefore indicate photosynthetic stress associated with alkane accumulation in thylakoid membranes. Interestingly, the remaining four other genes whose proteins were similarly upregulated by butachlor in three strains of *Anabaena* were also transcriptionally upregulated in the 2 g strain. The *N. punctiforme* homologues of these were: Npun_R0971, a HHE cation-binding protein; Npun_F3786, a signal transduction histidine kinase homologue containing four predicted transmembrane domains; Npun_F3789, a high light inducible protein; and Npun_R4582, a manganese-containing catalase homologue [48]. No similar parallels between gene expression in the 2 g strain and similar proteomic experiments testing for responses to oxidative stress were apparent [50]. As butachlor is a hydrophobic compound, these gene responses point to parallels between exposure to this herbicide and internal production of alkanes that may be unique to stress associated with hydrophobic compounds.

In addition to genes encoding potential structural and enzymatic proteins, several genes encoding regulatory proteins were also upregulated. In addition to SigB2 mentioned above, Npun_BF041, encoded on one of the five naturally occurring plasmids in *N. punctiforme,* belongs to the AraC family of transcriptional regulators and was found to be 8-fold upregulated in the 2 g strain. Two kinases were also identified; Npun_F1277 is a sensor signal transduction histidine kinase, and Npun_F2818 is an ABC1 kinase similar to ABC1K1 associated with *Arabidopsis thaliana* chloroplast plastoglobules. In *A. thaliana,* the latter regulates photosynthetic activity and photoprotection by controlling chloroplast tocopherol and plastoquinone production, and may be involved in integrating sugar/starch metabolism with photosynthetic processes [51]. The Npun_F2818 ABC1 kinase, as in plastoglobules, may be an LD-associated protein and be upregulated in response to the increased number of LDs in the 2 g strain. Only 26 bp downstream is Npun_F2819, similarly upregulated due to co-transcription with Npun_F2818 based upon RT-qPCR results (Table 2). Npun_F2819 has three predicted transmembrane domains, and when co-expressed with F2818, caused large decreases in the alkane content of whole cells and LDs, but only when present in a strain that already produced high levels of alkane (see below). These results indicate that Npun_F2818–2819 are potential negative regulators of alkane production.

In general, the putative roles of many upregulated genes products indicate that alkane overproduction is indeed stressful to cyanobacteria, and identifies potential novel adaptations to alkane stress. Based upon the large transcriptional increases, processing of rRNA required for ribosome assembly may be a target of alkane toxicity. Increased expression of genes also found after exposure of other (cyano)bacteria to a range of stress conditions may indicate general stress responses that overlap with alkane stress. These include those responsible for the production of mycosporine-glycine, a GsiB-like general stress-induced protein, multiple CsbD-like and HCP proteins, a BON-domain containing protein, and PRC-barrel proteins including AvaK. Novel adaptations to alkane overproduction suggested by this comparative transcriptomic study include accumulation of polyketides and/or non-ribosomal peptides, nostopeptolide A and a potentially internal microviridin-like cyclic peptide. Although not addressed in this study, future work will be required to determine if these compounds are indeed present in alkane-stressed cells, and their role in alkane tolerance in cyanobacteria. Identification of upregulated genes encoding a sigma factor, a DNA-binding protein and kinase proteins offers insights into potential regulatory elements involved in controlling the transcriptional response to alkane overproduction.

#### Downregulated genes in the alkane overproduction strain

Genes encoding proteins that may have direct effects on LD composition or abundance may be transcriptionally downregulated in the alkane overproducing strain in an attempt to restore the number of LDs to normal levels. Alternatively, downregulated genes could be in response to stress associated with excess alkane production, or simply a response to the slower growth rate exhibited by the 2 g strain.

Genes showing the largest decrease in the 2 g strain are photosynthetic, and include phycobilisome linker proteins and phorphyrin synthetic enzymes that are likely a response to the slower growth of the 2 g strain. Also, in this growth-related category are multiple subunits of NAD(P)H-quinone oxidreductase, reduction of several cell envelope synthesis proteins, as well as enzymes involved in amino acid biosynthesis, nucleic acid precursors and protein polymerization.

An example of a gene that is downregulated that may be directly involved in lipid droplet formation is Npun_F0518, a SpoVK vesicle-fusing ATPase with reduced expression in the 2 g strain. Reduced vesicle fusion could explain the high number of small LDs seen during exponential growth in the 2 g strain. Later, during stationary phase, these fuse into abundant large LDs, higher in abundance than in the control strain [10], indicating the fusion process is only delayed, but not eliminated, in the 2 g strain.

In the stress-associated gene category are three genes: Npun_R0404, one of the carotenoid-binding domain containing proteins that exhibited a large 11-fold downregulation in the 2 g strain; Npun_R0279, which contains a universal stress protein domain; and Npun_R4883, a low temperature-requirement A-like protein. This indicates that these proteins likely have a specialized function in the cell that is not required for dealing with alkane overproduction.

Regulatory proteins with reduced transcription in the 2 g strain include sigma factor SigC (Npun_F0996). This likely accounts for downregulation of the PII protein (GlnB; Npun_F4466), since this nitrogen-regulatory protein has been shown to be in the regulon of SigC [52]. The Npun_R1304 gene encoding a PadR-like transcriptional regulatory protein was also lower in the 2 g strain. Based on transcriptomic studies of other PadR-like repressors, this repressor protein may be responsible for regulating genes related to the cell envelope [53], and its 2.4-fold reduced expression may explain the increased transcription of several glycosyl-transferases and other envelope-related genes in the 2 g strain. Interestingly, downregulated plasmid genes were largely confined to plasmid D, one of the five naturally occurring plasmids in this strain, whereas upregulated genes were predominantly on plasmid B. Other plasmid-encoded genes include histidine kinases and putative DNA-binding proteins that may have effects on chromosomal gene transcription as well as plasmid-specific effects.

### Overexpression of selected genes and operons

To see if overexpression of genes that were upregulated in the comparative transcriptomic data resulted in enhanced alkane or LD production, 16 different multi-copy shuttle plasmids were constructed containing genes or operons alone (single gene), or in conjunction with the 3 g plasmid bearing Npun_F1710/1711/5141 to form four-gene (4 g) plasmids. The base plasmid chosen for this was the multi-copy shuttle plasmid pSCR119 that has 12–14 copies per genome. The 3 g plasmid was chosen for the second expression platform since it was previously shown to produce the highest quantities of alkanes when combined [10] and we wanted to see if additional genes could further boost production. The third gene in the 3 g plasmid, Npun_F5141, encodes a putative lipase that was hypothesized to increase free fatty acids and promote production of the fatty acyl-ACP substrate for the Aar/Ado enzymes. We hypothesized that if additional genes or operons found to be upregulated in the alkane-overexpressing strain were added to this 3 g plasmid to create 4 g plasmid-bearing strains, further increases in alkane production relative to whole-cell lipids would result. The resulting *N. punctiforme* set of plasmid-bearing strains, termed either single-gene or 4 g strains, were analysed for changes in their fatty acid and alkane content in both exponential and stationary phases of growth (Table 3). It should be noted that DNA fragments containing genes upregulated in this study sometimes contained several adjacent genes that could possibly be transcribed as an operon, but were classified as single-gene strains, or four-gene strains when added to the 3 g expression platform plasmids in Table 3, for simplicity.

**Table 3.** Percentage changes in area under the curve of FAME and alkane (C17) peaks produced during analysis by GC-MS of single and 4 g overexpressing strains relative to their respective controls. For single-gene strains, the control is the wild-type bearing pSCR119. For the 4 g strains (genes overexpressed in conjunction with Npun_R1710-1711 and Npun_F5141), the control is the 3 g strain. Numbers highlighted and in bold denote a *P*-value of <0.05

Single-gene OE								Four-gene OE							
**Whole-cell lipids, exponential**															
	C17	C16 : 1	C16 : 0	C18 : 2	C18 : 3	C18 : 1	C18 : 0		C17	C16 : 1	C16 : 0	C18 : 2	C18 : 3	C18 : 1	C18 : 0
Npun_R0971	1.8	−5.3*	8.2	−3.9	−9.7*	−3.2*	12.1*	Npun_R0971	−2.5	−0.1	0.9	−0.6	1.1	0.6	0.7
Npun_F1545	3.5	**−3.6**	4.3	1.0	**−8.4**	−2.8*	6.0	Npun_F1545	−8.1	0.7	**2.7**	−0.4	0.8	0.5	0.6
Npun_R5066	1.6	−1.6	**6.7**	−3.4	**−3.9**	−3.0*	3.6	Npun_R5066	−4.5	0.3	0.5	1.6	0.6	−0.5	**2.0**
Npun_R6442	2.7	−0.5	1.2	−1.7	−3.6	−0.3	2.2	Npun_R6442	2.3	−0.5	−1.7	0.2	−0.2	**−0.6**	0.4
Npun_F2189	2.2	−0.2	1.8	−1.7	−3.9	0.4	1.4	Npun_F2189	−1.3	**0.9**	0.5	0.2	−0.2	−0.1	0.0
Npun_F2818-19	1.9	2.1	−0.5	3.4	−3.9	**−1.2**	−1.9	Npun_F2818-19	**−9.2**	2.6*	1.7	0.8	**2.8**	0.5	0.8
Npun_F2191	0.4	0.4	2.4	−0.9	−3.4	0.2	0.8								
Npun_F2189-92	0.6	0.3	2.5	−0.7	−0.3	−0.5	−1.9								
Npun_R1848	0.0	−0.4	−0.5	1.5	−1.4	−0.3	1.2								
Npun_F5598−00	1.8	0.6	**−2.7**	1.0	−1.2	−0.1	0.6								
**Whole-cell lipids, Stationary**															
Npun_R0971	−1.0	0.8	−2.3*	0.7	−0.5	1.1*	1.2	Npun_R0971	−2.2	0.1	0.9	0.6	−0.3	0.3	0.6
Npun_F1545	**4.0**	−0.7	−2.2*	**−2.1**	−1.6*	1.8*	0.7	Npun_F1545	−3.2	−0.4	0.6	0.9	−0.2	0.2	0.4
Npun_R5066	−0.1	0.9	0.2	−2.2*	**−1.2**	0.1	2.3	Npun_R5066	−5.2	−0.9	1.1	1.4	1.1	0.3	2.2
Npun_R6442	−2.3	0.9	0.3	−0.9	−0.2	**0.2**	1.8	Npun_R6442	−13.7*	1	**3.3**	2.9*	2.3*	1.1*	**3.1**
Npun_F2189	0.3	−0.4	**−1.5**	−1.8*	−0.8	0.8*	**3.4**	Npun_F2189	**−10**	0	3.0	1.2	**1.7**	**0.6**	3.5*
Npun_F2818-19	−1.4	3.3*	−5.1*	0.9	3.3*	**0.5**	−1.6	Npun_F2818-19	**−13.9**	2.8*	2.8	**3.1**	**2.7**	**0.6**	2.0
Npun_F2191	0.2	2.2	−4.1*	1.7	−0.8	0.4	0.4								
Npun_F2189-92	1.4	1.4	0.2	−2.1	1.8	1.1*	−3.7*								
Npun_R1848	−1.9	−0.6	5.0*	−2.9	−0.1	0.0	0.5								
Npun_F5598−00	0.6	−0.3	0.3	**−3.1**	**1.2**	0.6*	0.7								
**LD lipids, exponential**															
Npun_F2191	0.9	0.0	−0.2	1.0	−0.3	0.0	−1.4	Npun_F2818-19	−15.3*	3.2*	5.8*	2.5*	1.9*	0.2	1.8
Npun_F2189-92	3.7	3.3*	−3.6	0.7	−0.7	0.0	−3.5								
**LD lipids, stationary**															
Npun_F2191	2.4	1.5	−2.6	1.0	0.0	0.6	−3.0	Npun_F2818-19	3.5	−1.7	−0.5	−1.1	−0.4	0.1	0.1
Npun_F2189-92	−5.5	−1.1	2.9	0.4	0.0	0.5	2.8								

*Denotes a *P*-value of <0.01.

#### Alkane and lipid profiles

By harvesting total lipids along with the co-extracted alkanes, and generating fatty acid methyl esters (FAMEs) for the lipids, we had a way of determining the relative amount of alkane production in these strains in addition to changes in fatty acid composition. FAME analysis of whole-cell lipids of several single-gene strains harbouring Npun_R0971, Npun_F1545, Npun_F5066 and Npun_F2818-19 showed a modest but significant decrease in unsaturated fatty acids during exponential phase. Of these, Npun_R0971 showed the largest alteration in fatty acid composition, with increased saturated C18 fatty acids and decreased unsaturated species. Npun_F5066 also increased the proportion of saturated fatty acids, but C16 : 0 rather than C18 : 0 was significantly affected in this strain. During stationary phase, these effects were mostly lost, and were replaced by many small, but significant, fatty acid changes in single-gene strains. It is important to note that the strain containing Npun_F2189-92 that does not produce stainable LDs, had decreased C18 : 0, the fatty acid precursor for synthesis of 17 carbon-long alkanes, the alkane type normally produced exclusively in this strain [1]. The only single-gene strain with increased alkane production harboured Npun_F1545, resulting in only a modest 4 % increased alkane accumulation during stationary phase.

Instead of further enhancing alkane production as anticipated, increased expression of several upregulated genes/operons in 4 g strains resulted in decreased alkane production. Among the 4 g strains, the only significant decrease in alkane production seen in exponentially growing cultures was that harbouring the Npun_F2818-19 operon, resulting in a 9.2 % decrease in alkane content relative to the control strain. During stationary phase, three 4 g strains had a significant reduction in alkane production. The Npun_F2818-19 strain continued to exhibit reduced alkane content, as did two additional strains harbouring Npun_R6442 and F2189, with stationary phase reductions in alkane content ranging from −10.0 to −13.9 % below controls (Table 3).

To see if LD composition paralleled the reduced alkane production observed in whole cells, LDs were isolated from these 4 g strains and compared to LDs from 3 g controls.

LDs from the 4 g Npun_F2818-19 strain in exponential phase showed a 15.3 % decrease in alkane content, paralleling the 9.2 % decrease in whole-cell alkane content under this growth condition. However, the large 13.9 % decrease in alkanes during stationary phase for whole cells was not apparent in stationary-phase LDs from the Npun_F2818-19 4 g strain, indicating that although the total cellular ratio of alkanes to total lipids was reduced in stationary phase, their proportion in LDs returned to normal. This indicates normal levels of the ABC1 protein kinase encoded by Npun_F2818, and/or the protein of unknown function encoded by Npun_F2819 may be required for proper alkane trafficking between LDs and cellular membranes during exponential growth, but not in stationary phase. There was an insignificant decrease in relative alkane to total lipid content in the LD-less Npun_F2189-92 single-gene strain, indicating that alkane production does not require the presence of LDs.

#### Phenotypic changes due to gene overexpression

**Fig. 2.**
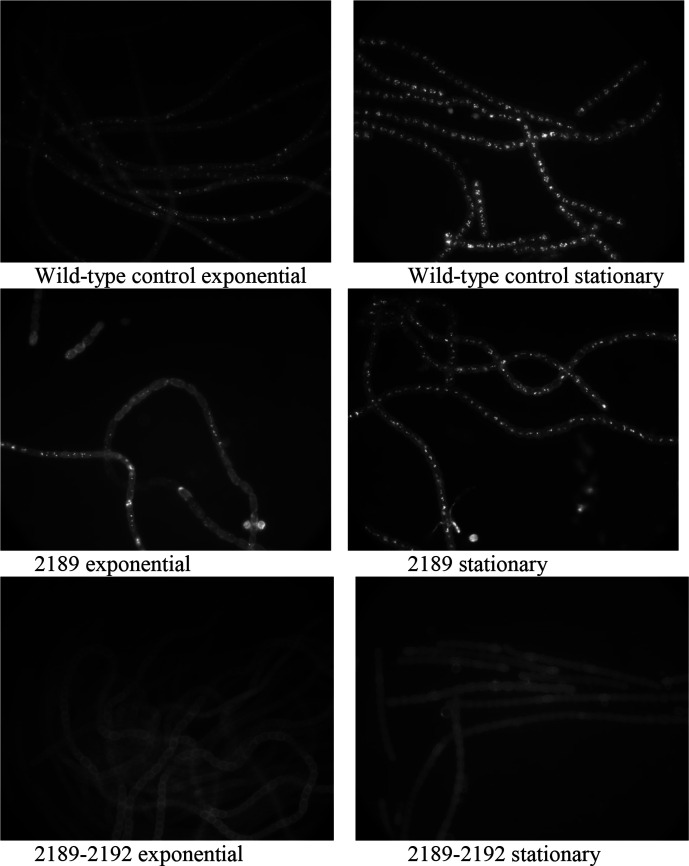
Fluorescent micrographs of BODIPY-stained strains of *N. punctiforme *.

Most single and 4 g overexpression strains tested did not produce any visible changes in LD size, localization, or abundance. However, the plasmid-bearing strain containing Npun_F2189 alone appeared to have BODIPY staining, indicative of neutral lipid rafts in the envelope, with reduced numbers of LDs during exponential phase (Fig. 2). When this DNA fragment was further extended to contain three additional upregulated genes, Npun_F2190, Npun_F2191 and Npun_F2192, this single-gene strain containing Npun_F2189-2192 exhibited no stainable LDs (Fig. 2). As discussed above, these encode structural genes with predicted or proven enzymatic function likely associated with microviridin production, not gene regulatory proteins, making it unlikely they directly repress genes associated with LD production. However, since microviridins can act as protease inhibitors, it may be possible that increased microviridin accumulation in this single-gene strain may increase the protein stability of regulatory proteins controlled by proteolysis.

**Fig. 3.**
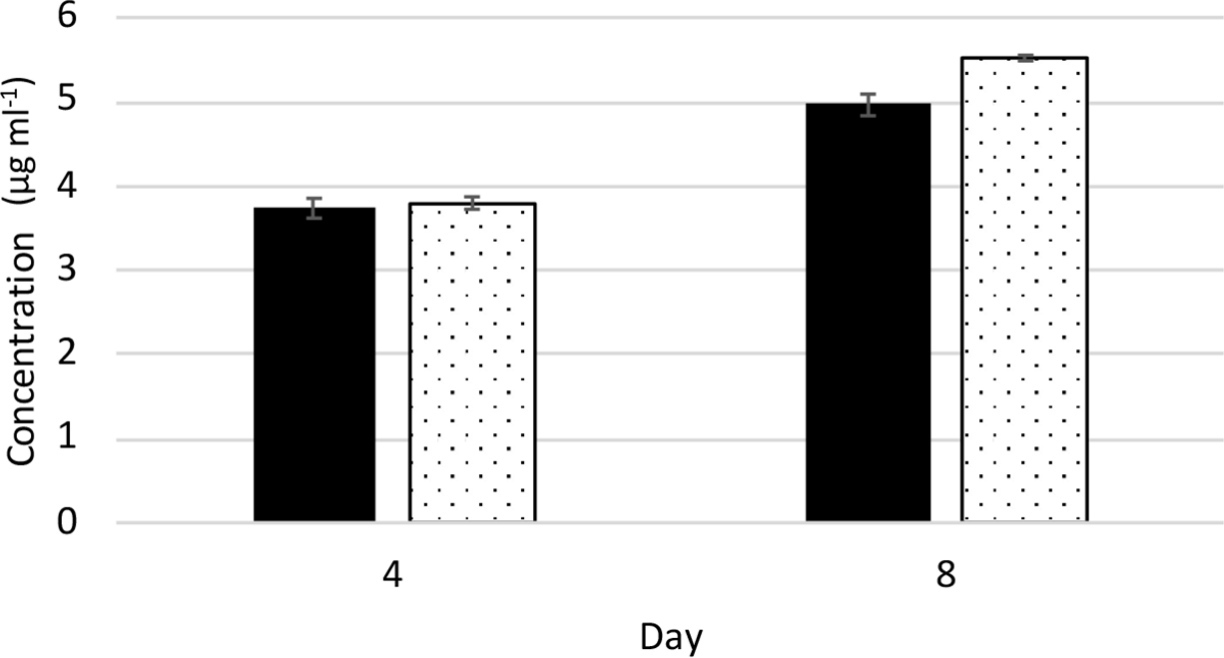
Growth of the strain bearing Npun_F2189-2192 after 4 and 8 days of high light (white bars) relative to a pSCR119 wild-type control (black bars). Chlorophyll a concentrations were determined from extractions from entire culture flasks to minimize sampling error. *n*=3, ±standard error.

Experiments were initiated to determine if there was a growth phenotype associated with the loss of LDs. The LD-less Npun_F2189-92-bearing strain exhibited identical growth rates to a wild-type control strain at both normal and cold temperatures under standard light conditions. Recently it was determined that cyanobacterial alkanes are required for normal photosynthetic cyclic electron flow, and growth at colder temperatures [9]. This study, however, used long-term selection for the mutation, so the phenotype might have been due to other secondary mutations. The work of Lea-Smith *et al*. [8], which quickly isolated a mutant in the same gene, exhibited similar photosystem II O_2_ production activity to the wild-type, but slower growth, increased cell size and division defects. Since the LD-less strain produced similar amounts of alkanes to the controls (Table 3), this could explain why no phenotypes were exhibited in earlier studies. To see if LDs that normally accumulate in stationary phase may be used to supply lipids for recovery from stationary phase, late stationary cultures of the LD-less and control strains were inoculated and monitored for alterations in lag phase recovery. No differences in lag phase between strains were detected, indicating that an alternative function exists. To see if LDs might be used to cope with high light stress, as has been suggested for plant chloroplast plastoglobules, the LD-less and control strains were subjected to high light (HL). The absence of LDs led to a small (11 %), but significant (*P*=0.014) increase in cell biomass after 8 days of HL that was not detected after only 4 days of HL (Fig. 3). There were no observable differences in bleaching after 8 days of HL, supporting our hypothesis that differences in growth rates resulted from a lack of LDs. This increased growth is likely due to relief of the metabolic drain on metabolites in the control strains used to form these inclusions. The physiological role of LDs therefore remains enigmatic, and we hope continued work in this area will elucidate their cellular function in the future. The identification of upregulated genes causing loss of LD production or reduced alkane production provides targets for future mutagenesis studies that we anticipate will increase alkane or LD production in cyanobacteria.

## Supplementary Data

Supplementary material 1
